# Impact of the COVID-19 pandemic on referral practices for pediatric central nervous system tumors: A Danish National Comparative Cohort Study

**DOI:** 10.1093/nop/npaf047

**Published:** 2025-04-28

**Authors:** Kathrine Synne Weile, René Mathiasen, Anne Sophie Lind Helligsoe, Lene Maria Ørts Clemmensen, Henrik Hasle, Louise Tram Henriksen

**Affiliations:** Department of Pediatrics and Adolescent Medicine, Aarhus University Hospital, Aarhus N, Denmark; Department of Oncology, Aarhus University Hospital, Aarhus N, Denmark; Department of Pediatric and Adolescent Medicine, Copenhagen University Hospital, Copenhagen, Denmark; Department of Clinical Medicine, Faculty of Medicine, University of Copenhagen, Copenhagen, Denmark; Department of Pediatrics and Adolescent Medicine, Aarhus University Hospital, Aarhus N, Denmark; Department of Pediatrics and Adolescent Medicine, Gødstrup Hospital, Herning, Denmark; Institute of General Medical Practice, Department of Public Health, HEALTH, Aarhus University, Aarhus, Denmark; Department of Pediatrics and Adolescent Medicine, Aarhus University Hospital, Aarhus N, Denmark; Department of Clinical Medicine, HEALTH, Aarhus University, Aarhus, Denmark; Department of Pediatrics and Adolescent Medicine, Aarhus University Hospital, Aarhus N, Denmark; Department of Clinical Medicine, HEALTH, Aarhus University, Aarhus, Denmark

**Keywords:** CNS tumors, children, COVID-19, diagnostic intervals, symptoms

## Abstract

**Background:**

Prolonged Diagnostic Interval (DI)s pose a challenge in childhood central nervous system (CNS) tumors. The coronavirus disease 2019 (COVID-19) pandemic altered contact with healthcare. The aim of this study was to describe DIs in children diagnosed with a CNS tumor in Denmark during the first year of the pandemic.

**Methods:**

We performed a retrospective questionnaire study, mapping time intervals and symptoms in the diagnostic pathway. We analyzed DIs measured in days and performed descriptive analyses of symptoms and contacts with health care professionals (HCP). Comparison to a pre-COVID cohort was applied. Intervals were presented as total diagnostic interval (TDI; time from symptom onset to diagnosis); Patient Interval (PI; time from symptom onset to first contact to an HCP); and DI (time from first contact to an HCP to diagnosis).

**Results:**

We included 25 patients (median age 8.2 years, 56% female). Median TDI: 98 days (IQR 26 210). The DI constituted the majority of TDI (median 70 days). Low-grade tumors displayed longer TDI than high-grade tumors. No significant difference in TDI was found when compared to a pre-COVID cohort (median 98 vs. 106 days). No hesitance to contact a doctor was reported by 88%, but 24% reported delays attributable to the pandemic. Patients reported more symptoms at onset, and the trajectory in the diagnostic pathways changed with fewer patients assessed by their general practitioner than pre-COVID.

**Conclusions:**

The DI was unaltered during the COVID-19 pandemic, but changes in trajectory were reported. Results stress the ongoing need for interventions to promote timely diagnosis.

**Key points**

1. total diagnostic intervals remained the same, during the first year of the pandemic.
2. Patients reported a greater number of symptoms at onset and more patients were diagnosed in an emergency setting, compared to a pre-COVID cohort
3. Pandemic or not, there is a need for interventions to promote timely diagnosis.

**Importance of the Study**

Diagnostic delay is a well-known challenge in diagnosing CNS tumors in children. Despite the small sample size and differences between the pre-COVID and COVID-cohort, this study confirms that the Danish healthcare system remained resilient during the pandemic, maintaining both incidence rates and Diagnostic Interval (DI)s for pediatric CNS tumors. At the same time, it underscores persistent social inequalities in time to diagnosis and highlights telemedicine’s growing impact. Ultimately, the findings emphasize the need for diagnostic decision support tools to further shorten DIs and address healthcare disparities.

In Denmark, tumors of the central nervous system (CNS) account for 20%–25% of malignancies in children with an age-standardized incidence of 42.1 per million person-years, resulting in approximately 50 new cases of children aged 0–17 years every year.^1^ CNS tumors are the leading cause of cancer-related death.^2,3^ Despite improved survival rates with a 5-year survival of 78%, long-term survivors face a substantial burden of late effects and reduced quality of life, due to tumor invasion and treatment, leading to acquired brain injury.^1,4,5^ Prolonged Diagnostic Interval (DI)s pose a well-established challenge. Presenting symptoms at onset vary by location, type of tumor as well as by age; symptoms are heterogeneous and are often mistaken as caused by more frequent and less serious disease.^6–8^

In 2020, the COVID-19 outbreak escalated into a worldwide pandemic presenting substantial challenges across the globe.^9^ In Denmark, the first lock-down was announced in March 2020. On a national basis, efforts to mitigate the spread were set into motion; citizens were urged to work from home, and schools and daycare closed. General practice closed for non-urgent consultations, and all patients were assessed by phone/video consultation before in-person appointments were scheduled. Within weeks, the practice changed, but all patients exhibiting symptoms resembling COVID-19 were only granted in-person contact following a negative COVID test result. Restricted closing of the country was put into effect on March 11th and gradual reopening started April 20th_,_ 2020. Though reopening, restrictions on assembly, social distancing measures, and other steps to contain the risk of infection were put into place. By winter, further restrictions were reinstated, and a second period of total lock-down was effective December 21st, 2020 through February 28th, 2021.^10,11^

In pediatric cancer communities, a reduced number of cases of solid and specifically CNS tumors were reported in the early pandemic,^12–14^ raising concern about prolonged DIs. It has been speculated that limited visits and reduced in-person assessment would affect diagnostic intervals, thus prolonging the time from symptom onset to set diagnosis (total diagnostic interval [TDI]).

Prior to launching hjernetegn.dk,^15^ an initiative to raise awareness on symptoms of childhood CNS tumors, a study was set up to investigate and map DIs and presenting symptoms in children aged 0–17 years with a CNS tumor diagnosed in a 5 year-period 2015–2019 in Denmark.^16^ The present study compares patients diagnosed within the first year of the pandemic to a pre-COVID cohort with the aim of providing a patient- and parent-reported clinical trajectory and presenting symptoms during the diagnostic process amid a global pandemic. We hypothesized that the DIs would be prolonged compared to a pre-COVID cohort due to implemented restrictions for contacting general practitioners (GPs) and hesitancy from parents and patients to contact their GP.

## Material and Methods

All incident cases of children aged 0–17 years diagnosed with a CNS tumor in Denmark 2015–2019 and the first year of the COVID-19 pandemic (March 11th, 2020 to March 10th, 2021) were identified and invited to participate in a questionnaire study. Data for the first 5-year period comprising 89 incident cases was analyzed and published separately, including a thorough description of methods.^16^ Questionnaires included information on early symptoms, dates of onset of symptoms, and contacts to the healthcare system including the role of the GP and mother's educational level as a proxy for socioeconomic status (SES). For the COVID-19 cohort, a series of questions regarding hesitance and waiting times due to the pandemic were added.

With approval from the Danish Data Protection Agency (file number: 1-16-02-300-19) through the Danish Health Data Authority, we applied for contact information on all Danish patients aged 0–17 years at the time of diagnosis. In accordance with the International Classification of Diseases 10th revision (ICD-10),^17,18^ diagnoses were defined as ICD-10: DC69-72, DC751-753, DD32-33, DD352-354, DD42-4, or DD443-DD445 in the Danish National Patient Register^19^ and the Civil Registration System. Validation of histological diagnosis, tumor location, and date of diagnosis was done through the Danish Childhood Cancer Registry (DCCR), a nationally validated clinical quality database.^20^ When reporting, tumor grade was grouped as low-grade or high-grade. Low grade is defined as WHO 1–2 and high-grade WHO 3–4. The low-grade tumor group comprised choroid plexus tumor, pilocytic astrocytoma, optic glioma, diffuse astrocytoma, other/not specified astrocytoma, craniopharyngioma, mixed glial-neuronal tumor, ganglioglioma, schwannoma, meningioma. High-grade tumors comprised ependymoma, glioblastoma, anaplastic astrocytoma, pleomorphic xanthoastrocytoma, medulloblastoma, mixed and unspecified glioma, atypical teratoid/rhabdoid tumor (AT/RT), and germinoma, in accordance with International Classification of Childhood Cancer, third edition (ICCC-3).^21^ Location of tumors was grouped as infratentorial (cerebellum/4th ventricle and brainstem) or supratentorial (cerebrum, supratentorial central area, hypothalamic, optic nerve and chiasm, and pineal gland), and spinal.

**Figure 1.**
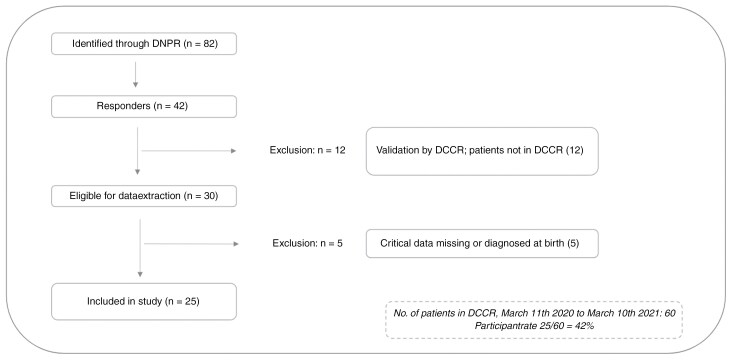
Inclusion flowchart.

Questionnaires were set up in Research Electronic Data Capture (REDCap)^22^ and sent directly to patients/parents through the National Digital Post. Questionnaires were sent out in March 2022 and inclusion ended in August 2022. For patients below the age of 18 years at the time of conducting the study, the custody-holding parent(s) were invited to participate. The flow of inclusion is shown in Figure 1. Invitations were re-sent to non-responders; due to legislation and GDPR, it was not possible to retrieve data on non-responders for analysis and comparison.

A total of 25 participants were included in the analyses. According to data from the DCCR, 60 patients were diagnosed within the first year of the pandemic approaching a participation rate of 42%. The number of monthly diagnosed patients in the first year of the pandemic is visualized in Figure S1.

In Denmark, access to health care is free and covered through taxes. The primary sector in Danish health care comprises practicing specialty physicians and GPs. The GP is the entry point to the health care system as access to specialty doctors (except ophthalmologists and ear-nose-throat) requires referral from the GP. The secondary sector comprises hospitals and emergency care.^17^

Responders provided the date of symptom onset and first contact with a healthcare professional (HCP). The date of set diagnosis by neuroimaging was obtained from the DCCR. TDI was defined as the time from symptom onset to set diagnoses. TDI was divided into Patient Interval (PI) and Diagnostic Interval (DI). PI was defined as the interval between the date of onset of symptoms and the first presentation to a health care professional (HCP). DI was defined as the interval between the date of the first presentation to an HCP and to date of the set diagnosis. The definition of DIs is modified from the Aarhus Statement.^23,24^

### Statistics

Descriptive analyses were performed analyzing time intervals to measure DIs in days; TDI, PI, and DI (median; interquartile interval (IQR)). Medians with 25 and 75 percentiles were presented, and differences between groups were tested using the Wilcoxon rank-sum, Kruskal–Wallis, and chi-square when suited. *P* < .05 was considered statistically significant. All statistical analyses were performed using the Stata/SE 18.0.

## Results

### Patient and Tumor Characteristics

Patient and tumor characteristics are shown in Table 1. A total of 25 patients were included for further analyses. Median age at diagnosis was 8.2 years (range 10.8 months to 16.8 years). The sex ratio (female:male) was 1:0.79, 56% (*n* = 14) were female.

**Table 1.** Patient and Tumor Characteristics

Patient and tumor characteristics		
All patients		25(100%)
Sex (*N* [%])	F	14 (56%)
	M	11 (44%)
Age at diagnosis, median (IQR)		8.2 (4.6, 13.5)
Age group at diagnosis (*N* [%])	0–4 years	7 (28%)
	5–9 years	6 (24%)
	10–14 years	10 (40%)
	15–17 years	2 (8%)
Tumor grade (*N* [%])	Low grade (WHO 1–2)	21 (84%)
	High grade (WHO 3–4)	4 (16%)
Tumors by location, grouped (*N* [%])	Infratentorial	8 (32%)
	Supratentorial	13 (52%)
	Spinal	4 (16%)
Diagnostic intervals in days, median (IQR)	Total interval	98 (25, 210)
	Patient interval	14 (5, 44)
	Diagnostic interval	70 (15, 184)

Patient and tumor characteristics. IQR, Interquartile range; Low Grade: choroid plexus tumor, pilocytic astrocytoma, optic glioma, diffuse astrocytoma, other/not specified astrocytoma, craniopharyngioma, mixed glial-neuronal tumor, ganglioglioma, schwannoma, meningioma; High-grade: ependymoma, glioblastoma, anaplastic astrocytoma, pleomorphic xanthoastrocytoma, medulloblastoma, mixed and unspecified glioma, atypical teratoid/rhabdoid tumor (AT/RT) and germinoma, in accordance with ICCC-3.^*21*^

Histological diagnosis was available for 22 patients, and 3 patients were diagnosed solely by neuroimaging. The most frequent tumors were pilocytic astrocytoma accounting for 44%. Most tumors were low-grade (84%) and most often supratentorial (52%). The distribution of tumor location by age differed significantly (*P* = .044), but not by tumor grade (*P* = .52). Frequency of tumors by grade, location, and age were skewed compared to the national average, where tumors are most frequent in patients aged 0–4 years.^1^

### Diagnostic Intervals

The reported TDI showed a range of 0–4489 days with a median of 98 days (IQR 25,210). The median PI was 14 days (IQR 5,44; range 0–365 days) and the median DI was 70 days (IQR 15,184; range 0–4489 days). Five patients displayed a TDI exceeding 365 days. Twenty-five patients were included for analysis, but data for sub-analysis differs within groups. Table 2 shows TDI, PI, and DI by age, tumor grade and location, number of symptoms at onset, contact to HCP and diagnosis, number of visits to GP prior to diagnosis, and mother's education as a proxy for SES. Low-grade tumors showed a significantly longer DI than high-grade tumors (*P* = .049). No statistical difference was seen between the sexes. Within age groups, patients aged 15–17 years displayed the longest intervals, but due to the small sample size (*n* = 2), they were excluded from further analysis. In the remainder, patients aged 0–4 years displayed the longest TDI, whilst patients aged 10–14 years reported the longest DI (median 78 days; IQR 47,383). PIs contributed the least to the TDI but were longest in the age of 0–4 years and 10–14 years with a median in both groups of 14 days. Low-grade tumors displayed a longer TDI than high-grade tumors (median 148 vs 60 days). By number of presenting symptoms at onset, patients presenting 4 or more symptoms displayed the longest TDI and DI, but the shortest PI. Regarding the number of symptoms present at first contact with an HCP, patients reporting 2–3 symptoms displayed the longest TDI (median 231 days) and the longest DI (231 days); PI was significantly shorter in patients presenting most symptoms (*P* = .045). There was no significant difference in SES, but results show mother's attended educational years were inversely proportional to the length of TDI, with the shortest TDI, PI, and DI in patients where the educational level was the highest. Patients with symptom onset within the first year of the pandemic were analyzed by month of onset displaying a TDI of the median of 52 days if diagnosed in the first 3 months of the pandemic, and a TDI of the median of 25 days if diagnosed in the latter part of the year (*P* = .58).

**Table 2.** Diagnostic Intervals By Sex, Age, Tumor Grade, Location, Symptoms, and Visits to GP

	*N*	Total interval, median (IQR), days	*P*-value	Patient interval, median (IQR), days	*P*-value	Diagnostic interval, median (IQR), days	*P*-value
All patients							
	25	98 (25, 210)		14 (5, 44)		70 (15, 184)	
Sex							
F	14	98 (74, 210)		18 (5, 83)		70 (20, 149)	
M	11	98 (20, 231)	.57	9 (0, 14)	.10	51 (6, 231)	.81
Age at diagnosis							
0–4 years	7	148 (19, 231)		14 (5, 16)		132 (6, 231)	
5–9 years	6	60 (20, 84)		11.5 (7, 61)		20 (0, 70)	
10–14 years	10	102 (74, 383)	.40	14 (4, 44)	.90	78 (47, 383)	.20
15–17 years	2	578		224		354	
Tumor grade							
Low grade	21	148 (25, 231)		14 (5, 31)		85 (20, 231)	
High grade	4	60 (28, 91)	.18	31 (11, 65)	.35	18 (3, 41)	*.049*
Location of tumor							
Supratentorial	13	98 (19, 231)		14 (4, 44)		70 (6, 231)	
Infratentorial	8	116 (29, 182)		13 (6, 72)		50 (13, 141)	
Spinal	4	141 (55, 534)	.86	14 (10, 23)	.95	127 (45, 512)	.64
Number of symptoms present at onset							
1 symptom	6	87 (25, 98)		46 (13, 83)		34 (1, 51)	
2–3 symptoms	6	127 (84, 1003)		15 (9, 20)		109 (70, 638)	
4 or more symptoms	13	177 (20, 231)	.42	7 (4, 14)	.22	149 (15, 231)	.11
Number of symptoms present at first contact to HCP							
1 symptom	12	95 (23, 182)		46 (11, 83)		49 (6, 117)	
2–3 symptoms	5	231 (148, 870)		14 (0, 16)		231 (132, 839)	
4 or more symptoms	8	56 (20, 198)	.13	7 (5, 14)	*.045*	50 (11, 174)	.08
Number of visits to GP prior to referral							
No visits	6	44 (13, 148)		6 (0, 13)		38 (1, 132)	
1 visit	6	134 (84, 1003)		14 (5, 44)		116.5 (47, 638)	
2–3 visits	5	105 (98, 198)		20 (14, 31)		85 (51, 184)	
4 or more visits	2	221	.26	31	.27	190	.39
Mother’s education as proxy for SES							
<13 years	5	198 (83, 210)		61 (14, 83)		149 (15, 184)	
13–15 years	14	127 (20, 383)		14 (4, 20)		78 (11, 383)	
>15 years	6	64 (25, 98)	.56	7 (5, 44)	.28	39 (20, 51)	.62
Patients with symptom onset the first year of COVID-19, grouped by months of onset							
March 2020–June 2020	6	52 (20, 105)		14 (9, 20)		13 (5, 85)	
July 2020–March 2021	5	25 (14, 98)	.58	13 (5, 47)	.71	20 (1, 51)	.78

Diagnostic intervals at a glance. IQR, Interquartile range; HCP, Healthcare professional; GP, General practitioner; SES, Socioeconomic status. Italics indicate statistical difference, with *P* < .05. Analysis in patients aged 15–17 and patients with 4 or more visits to a GP prior to diagnosis, were not disclosed due to sample size.

### Presenting Symptoms at Onset and by Time of Diagnosis

Patients reported symptoms from a list of 27 items (Table S1). More than 75% of patients reported 2 or more symptoms present and 52% reported 4 or more symptoms present at the time of onset. At the first presentations to an HCP, 46% reported one symptom, and 35% reported 4 symptoms or more. At diagnosis, 62% presented 4 or more symptoms. The most frequent symptoms at onset and at first presentation to an HCP were headache (44% and 40%), vomiting (36% and 24%), nausea (32% and 20%), and visual symptoms (32% and 24%); at diagnosis the 3 most frequent symptoms were headache (52%), visual symptoms (40%) vomiting and nausea (32%) and unspecified pain (32%). Patients reported no fever, signs of infection, visible tumor or lump, early puberty, or diabetes insipidus at onset. Table S2 shows presenting symptoms at the time of onset.

### Hesitation and Delays Caused by the Pandemic

In the questionnaire items regarding hesitation or postponement due to the pandemic, 88% of patients reported no hesitation in contact with their doctor. Hesitation was defined as self-reported reluctance to contact an HCP. Table 3 shows reported hesitance to contact a doctor, the time from contact to actual assessment in consultation, and type of consultation. Twenty-four percent experienced delays due to the pandemic. Table S3 shows a sub-analysis of patient- and parent-reported first contacts and type of consultation.

**Table 3.** First Contact and Consultation

	All patients	
*N*	25	%
Did you hesitate to contact a doctor due to the pandemic?		
No	22	88%
Yes	2	8%
How long did you postpone contacting a doctor?		
0 days	12	48%
1–3 days	2	8%
4–7 days	1	4%
8–14 days	1	4%
15–21 days	1	4%
22 days or more	1	4%
Do not recall	3	12%
From the time you made contact, how much time passed before assessment?		
0 days	8	32%
1–3 days	5	20%
4–7 days	3	12%
8–14 days	2	8%
Do not recall	4	16%
How was the initial consultation conducted?		
Consultation and assessment by phone	4	16%
Clinical consultation	14	56%
Other (eg, video consultation)	5	20%
Did you experience delays or cancellations regarding in the early trajectory?		
No	18	72%
Yes	6	24%

Questionnaire items regarding patient or parental behavior, regarding first contact with an HCP.

Responders were given the option to comment in the open text. Qualitative responses included the following statements:

#### Parent statement 1.—

“At the time of diagnosis, it coincided with the beginning of the lock-down. Everything was difficult, and we were allowed to have only one parent present [in the hospital], and the recommendations changed daily. It was a stress factor. Our family doctor had to fight to ensure that [patient] was seen at the local hospital.”

#### Parent statement 2.—

“Our appointment at the pediatric clinic was moved forward due to severity, as other [less severe] appointments were canceled because of COVID-19.”

#### Parent statement 3.

—“…in the end, we got a referral for a CT scan, but there was a long wait due to COVID.”

### Trajectory in Clinical Course of Events

Mapping the pathway, data was available on all patients but one (*n* = 24). Fifty-two percent (*n* = 13) attended a GP as their first contact with an HCP; 10 patients reported being assessed by their own GP. Nine patients (36%) were assessed in out-of-hours GP or in an emergency setting (pediatric department or a broad emergency department). The remainder (*n* = 2) were assessed by specialty doctors. In reporting the number of visits to the GP prior to referral to a specialist or scan, data was available for 19 patients, where 24% (*n* = 6) reported not involving their GP, 24% visited their GP only once (*n* = 6) and 28% (*n* = 7) reported 2 or more visits with their GP. A trend showed that patients assessed at their own GP displayed the longest DI with a median of 117 days (IQR 51,184) and patients assessed in out-of-hours GP or pediatric department displayed the shortest DIs with a median of 7.5 days (IQR 0,15) and 10 days (IQR 0,20), respectively. No statistically significant differences were found.

### DIs compared With Children Diagnosed From 2015 to 2019

Patient characteristics of all patients diagnosed within the first year of the pandemic were compared to a pre-COVID cohort comprised of patients diagnosed in the 5-year period 2015–2019. No statistically significant differences occurred between the 2 groups (COVID vs pre-COVID) regarding sex, age, DI, symptoms at onset and first contact with HCP, and number of visits to GP prior to referral or SES.

Low-grade tumors constituted 84% of cases in the COVID-cohort, but only 55% in the pre-COVID cohort. A trend was found regarding the number of presenting symptoms, which was higher at the time of onset in the COVID cohort reporting 4 or more symptoms in 52% (*n* = 13) versus 39% (*n* = 35) in the pre-COVID cohort (*P* = .34). Addressing the trajectory on the same note, in the pre-COVID cohort 63% attended their own GP as first HCP, while this only applied to 40% in the COVID cohort. A comparison of patient characteristics in detail is shown in Table 4.

**Table 4.** Patient Characteristics for Comparison, COVID and 2015–2019-Cohort

		COVID	2015–2019	*P*-value
		*N*(%)	*N*(%)	
All patients		25(100%)	89(100)	
Sex	F	14 (56%)	41(46%)	.38
	M	11 (44%)	48 (54%)	
Age at diagnosis, median (IQR)		8.2 (4.6, 13.5)	7 (2.6,10.9)	.14
Age group at diagnosis	0–4 years	7 (28%)	35(39%)	.66
	5–9 years	6 (24%)	21(24%)	
	10–14 years	10 (40%)	25 (28%)	
	15–17 years	2 (8%)	8 (9%)	
Tumor grade, WHO	Low grade (WHO 1–2)	21 (84%)	49 (55%)	*.02*
	High grade (WHO 3–4)	4 (16%)	30 (34%)	
	Unknown	—	10 (11%)	
Tumors by location	Infratentorial	8 (32%)	46 (52%)	*.04*
	Supratentorial	13 (52%)	36 (41%)	
	Spinal	4 (16%)	3 (3%)	
	Unknown	—	4 (4%)	
Diagnostic intervals, median (IQR)	Total interval	98 (26, 210)	106 (26, 227)	.59
	Patient interval	14 (5, 44)	12 (0, 31)	.46
	Diagnostic interval	70 (15, 184)	50 (7, 165)	.50
Mother’s attended education, years	<13 years	5 (20%)	16 (18%)	.89
	13–15 years	14 (56%)	49 (55%)	
	>15 years	6 (24%)	22 (25%)	
	Missing	—	2(2%)	
Symptoms present at symptom onset	1 symptom	6 (24%)	21 (24%)	.42
	2–3 symptoms	6 (24%)	33 (37%)	
	4 or more symptoms	13 (52%)	35 (39%)	
Symptoms present at first contact to HCP	No symptoms	0 (0%)	2 (2%)	.30
	1 symptom	12 (48%)	30 (34%)	
	2–3 symptoms	5 (20%)	33 (38%)	
	4 or more symptoms	8 (32%)	22 (25%)	
Symptoms present at diagnosis	No symptoms	0 (0%)	4 (5%)	*<.01*
	1 symptom	9 (36%)	10 (12%)	
	2–3 symptoms	1 (4%)	24 (30%)	
	4 or more symptoms	15 (60%)	42 (52%)	
Number of visits to GP prior to referral	No visits	6 (24%)	16 (18%)	.65
	1 visit	6 (24%)	30 (34%)	
	2–3 visits	5 (20%)	18 (20%)	
	4 or more visits	2 (8%)	12 (13%)	
First attended HCP	GP (own family doctor)	10 (40%)	55 (63%)	.45
	Other GP (eg, temp)	3 (12%)	9 (10%)	
	Out-of-hours GP	2 (8%)	4 (5%)	
	Emergency dept.	5 (20%)	6 (7%)	
	Pediatric dept.	2 (8%)	4 (5%)	
	Specialty doctor, practice	2 (8%)	4 (5%)	

Patient characteristics for all patients diagnosed during the first year of COVID-19 and from 2015 to 2019. Italics indicate statistically significant differences, *P* < .05. IQR, Interquartile range.

## Discussion

The COVID-19 pandemic significantly disrupted healthcare systems globally. In Denmark, despite these challenges, our study found that the total number of pediatric CNS tumors diagnosed in the first year of the pandemic (*n* = 60) remained consistent with pre-pandemic incidence rates.^1^ Additionally, the median time to diagnosis interval (TDI) was 98 days during the pandemic, showing no statistically significant difference compared to the pre-pandemic median of 106 days (*P* = .59; Table 4).^16^ In a sub-analysis of patients whose symptom onset occurred during the first 3 months of the pandemic (March 10–June 2020), the median TDI was 52 days. This subgroup was small, highly selected, and biased toward patients whose symptoms and diagnosis fell within the same calendar year. As a result, no definitive conclusions or trends can be drawn from this subset analysis.

Our findings align with findings from other studies. In our neighboring country, Norway, Jarvis et al. reported stable numbers of CNS and solid tumor diagnoses during the pandemic in 2020.^25^ Another recent publication with data from the Central Brain Tumor Registry of the United States indicated a temporary decrease in CNS tumor incidence during the initial months of the pandemic, primarily driven by nonmalignant tumors.^14^ Moreover, Offenbacher et al. observed a decrease in solid tumor diagnoses in New York during the first lock-down month followed by a subsequent increase.^26^

The median TDI of 98 days, with more than half of the patients experiencing increased DIs exceeding 3 months, aligns with pre-pandemic data but is notably longer than some comprehensive studies reporting TDIs ranging from 28 to 123 days.^8,27–43^ This discrepancy may stem from differences in data collection methods, patient populations, or healthcare system responses. Low-grade tumors, which typically have slower growth rates, and may have more subtle symptoms depending on the localization of the tumor, were associated with longer DIs, an observation also supported by other studies.^32,42^

In countries with educational interventions intended to decrease the DI, the impact of the COVID-19 pandemic did not seem to affect DIs significantly. Canova et al. published a study in 2023 describing the effects of educational interventions and the COVID-19 pandemic on time to diagnosis in pediatric patients with a CNS tumor in the United States.^44^ Within the COVID population, findings indicated no significant difference in TDI compared to a historic cohort. Williams et al. made the same conclusion in a multi-center evaluation of newly diagnosed cancers in children during the pandemic in the United Kingdom.^45^ The Danish CNS tumor awareness initiative hjernetegn.dk was launched post-pandemic in August 2023.^15^

During the pandemic, participants reported a higher number of symptoms at onset and at diagnosis, with 52% and 60% experiencing more than 4 symptoms, respectively. Notably, only 40% attended their own general practitioner (GP) as the first healthcare provider compared to 63% in the pre-pandemic cohort. Efforts to mitigate spread undoubtedly had a major impact, including societal consequences such as structural changes in the contact with both the primary and secondary sectors, thereby impacting both HCPs and the general population. The increased reliance on emergency departments (20%) suggests a shift in referral practices, potentially due to GP closures and the redirection of patients to telemedicine or testing centers. This change may indicate hesitancy or prolonged PIs, despite only 8% of parents reporting hesitancy to contact healthcare services. For comparison, a study from Italy reported prolonged DIs as a result of fear in the early phase of the pandemic.^46^

Our study identified social inequalities affecting DIs. Children with lower parental education levels experienced significantly longer TDIs (median 198 days) compared to those with higher parental education (median 64 days). This finding aligns with a Dutch study highlighting socioeconomic status disparities in non-COVID-related healthcare utilization during the pandemic.^47^ Addressing these inequalities is crucial to ensure equitable healthcare access and timely diagnosis for all patients.

The pandemic has expanded the use of telemedicine; a use that is here to stay. While telemedicine has certain limitations such as the absence of physical examination and direct interpersonal interaction, it has been acknowledged that it offers a notable advantage in terms of time efficiency for both patients and healthcare facilities.^48^ Shawwa et al. (2023) have emphasized the importance of incorporating telemedicine into medical education, thereby equipping future healthcare professionals with the necessary skills to engage in this form of communication.^49^ This would also educate HCPs on potential inequalities and disparities when implementing eHealthcare.

A review by Katato et al. 2023^50^ discusses impact and learnings in the pediatric oncology community, concluding that outcomes were not as severe as feared, but the impact on diagnostics and management of pediatric patients with malignancies was affected, primarily in a hospital setting. Applied to a Danish setting in the secondary sector patients were managed as usual. The results of this study indicate that even though lock-down and perceived fear changed primary health care, it did not result in increasing DIs. While intervals remain unchanged, so do the posing challenges of timely diagnosis in CNS tumors.

### Strengths and Limitations

This study benefits from its national scope and inclusion of patient-reported data, providing valuable insights into the diagnostic journey of pediatric CNS tumor patients during the pandemic. However, limitations include potential recall and selection biases inherent in self-reported data, a small sample size that may not fully represent national incidence distributions and differences in age and tumor characteristics compared to pre-pandemic cohorts. Future studies should consider prospective data collection to minimize these biases to enhance generalizability.

Further research should explore the long-term impacts of pandemic-induced healthcare changes on diagnostic processes and outcomes in pediatric CNS tumors. Developing and implementing diagnostic decision support tools could enhance early detection and reduce DIs. Additionally, addressing social inequalities in healthcare access remains a priority to ensure timely and equitable care for all patients.

## Conclusion

Overall, our findings indicate that DIs were not prolonged during the pandemic. However, our study shows a change in trajectory, suggesting that steps to mitigate the pandemic posed a challenge in an already challenging diagnostic process. The learnings from this study underline the need for diagnostic decision support tools to accelerate the early diagnostic process in childhood CNS tumors.

## Supplementary Material

npaf047_suppl_Supplementary_Materials

## Data Availability

The data that support the findings in this study can be obtained on request from the corresponding author. Due to ethical restrictions and Danish legislation, data is not publicly available.
