# Supplementary material for: Impact of the COVID-19 pandemic on referral practices for pediatric central nervous system tumors: A Danish National Comparative Cohort Study
Source: Neurooncol Pract. 2025 Apr 28;12(5):912–21. doi: 10.1093/nop/npaf047 (PMC12508746; doi:10.1093/nop/npaf047)
Supplement: npaf047_suppl_Supplementary_Materials [file npaf047_suppl_supplementary_materials.docx]

SUPLEMENTAL MATERIAL

Supplemental figure S1: Number of patients diagnosed within the first year of the pandemic.

##

*Figure 1: Frequency of patients diagnosed with a tumor in CNS in Denmark during the first year of the pandemic, according to Danish Childhood Cancer Registry (DCCR). N=60.*

Supplemental table S1: Symptoms at onset, first contact to HCP and at time of diagnosis.

|  | Onset | | | | First contact to HCP | | | | Diagnosis | | | |
| --- | --- | --- | --- | --- | --- | --- | --- | --- | --- | --- | --- | --- |
|  | COVID | | 2015-2019 | | COVID | | 2015-2019 | | COVID | | 2015-2019 | |
| Total | 25 | % | 89 | % | 25 | % | 89 | % | 25 | % | 89 | % |
| Headache | 11 | 44% | 41 | 46% | 10 | 40% | 40 | 45% | 13 | 52% | 44 | 49% |
| Vomiting | 9 | 36% | 40 | 45% | 6 | 24% | 34 | 38% | 8 | 32% | 39 | 44% |
| Nausea | 8 | 32% | 24 | 27% | 5 | 20% | 18 | 20% | 8 | 32% | 28 | 31% |
| Visual symptoms | 8 | 32% | 22 | 25% | 6 | 24% | 15 | 17% | 10 | 40% | 25 | 28% |
| Pain, unspecified | 7 | 28% | 17 | 19% | 5 | 20% | 16 | 18% | 8 | 32% | 24 | 27% |
| A feeling of something wrong | 6 | 24% | 31 | 35% | 5 | 20% | 18 | 20% | 6 | 24% | 21 | 24% |
| Pallor | 5 | 20% | 8 | 9% | 3 | 12% | 6 | 7% | 4 | 16% | 13 | 15% |
| Dizziness | 5 | 20% | 16 | 18% | 4 | 16% | 11 | 12% | 6 | 24% | 13 | 15% |
| Behavioral change | 4 | 16% | 7 | 8% | 3 | 12% | 4 | 4% | 4 | 16% | 8 | 9% |
| Abnormal head position | 4 | 16% | 6 | 7% | 2 | 8% | 5 | 6% | 4 | 16% | 7 | 8% |
| Drowsiness or fatigue | 4 | 16% | 19 | 21% | 3 | 12% | 14 | 16% | 6 | 24% | 30 | 34% |
| Loss of appetite | 4 | 16% | 12 | 13% | 4 | 16% | 5 | 6% | 4 | 16% | 13 | 15% |
| Weight loss | 4 | 16% | 8 | 9% | 2 | 8% | 6 | 7% | 5 | 20% | 16 | 18% |
| Seizures | 4 | 16% | 6 | 7% | 4 | 16% | 5 | 6% | 4 | 16% | 6 | 7% |
| Other symptoms, not specified | 4 | 16% | 6 | 7% | 4 | 16% | 8 | 9% | 5 | 20% | 1 | 1% |
| Balance/co-ordination problems) | 3 | 12% | 19 | 21% | 3 | 12% | 12 | 13% | 7 | 28% | 23 | 26% |
| Sudden numbness/loss of function in extremities | 2 | 8% | 5 | 6% | 2 | 8% | 1 | 1% | 5 | 20% | 7 | 8% |
| Abnormal eye movements | 1 | 4% | 6 | 7% | 0 | 0% | 8 | 9% | 2 | 8% | 6 | 7% |
| Abnormal growth | 1 | 4% | 3 | 3% | 0 | 0% | 2 | 2% | 0 | 0% | 2 | 2% |
| Delayed development of motor skills | 1 | 4% | 6 | 7% | 0 | 0% | 5 | 6% | 2 | 8% | 8 | 9% |
| Fever | 0 | 0% | 5 | 6% | 0 | 0% | 4 | 4% | 0 | 0% | 5 | 6% |
| Signs of infection (other than fever) | 0 | 0% | 0 | 0% | 0 | 0% | 1 | 1% | 0 | 0% | 1 | 1% |
| Visible tumor/lump/swelling | 0 | 0% | 1 | 1% | 0 | 0% | 1 | 1% | 0 | 0% | 0 | 0% |
| Increasing head circumference | 0 | 0% | 7 | 8% | 0 | 0% | 3 | 3% | 1 | 4% | 10 | 11% |
| Facial palsy | 0 | 0% | 1 | 1% | 0 | 0% | 0 | 0% | 3 | 12% | 2 | 2% |
| Early puberty | 0 | 0% | 1 | 1% | 0 | 0% | 0 | 0% | 0 | 0% | 1 | 1% |
| Delayed puberty | 0 | 0% | 1 | 1% | 0 | 0% | 0 | 0% | 1 | 4% | 0 | 0% |
| Diabetes Insipidus | 0 | 0% | 1 | 1% | 0 | 0% | 1 | 1% | 0 | 0% | 2 | 2% |

*Table S1: Overview of symptoms at onset, first contacts to a HCP and at time of diagnosis. COVID covers reulsta from patients diagnosed during the first year of COVID-19; 2015-2019 covers patients diagnosed within that time period. HCP: Healthcare professional.*

Supplemental table S2: Presenting symptoms

| Table 3 | Onset | | | | First contact to HCP | | | | Diagnosis | | | |
| --- | --- | --- | --- | --- | --- | --- | --- | --- | --- | --- | --- | --- |
|  | COVID | | 2015-2019 | | COVID | | 2015-2019 | | COVID | | 2015-2019 | |
| Total | 25 | % | 89 | % | 25 | % | 89 | % | 25 | % | 89 | % |
| Headache | 11 | 44% | 41 | 46% | 10 | 40% | 40 | 45% | 13 | 52% | 44 | 49% |
| Vomiting | 9 | 36% | 40 | 45% | 6 | 24% | 34 | 38% | 8 | 32% | 39 | 44% |
| Nausea | 8 | 32% | 24 | 27% | 5 | 20% | 18 | 20% | 8 | 32% | 28 | 31% |
| Visual symptoms | 8 | 32% | 22 | 25% | 6 | 24% | 15 | 17% | 10 | 40% | 25 | 28% |
| Pain, unspecified | 7 | 28% | 17 | 19% | 5 | 20% | 16 | 18% | 8 | 32% | 24 | 27% |
| A feeling of something wrong | 6 | 24% | 31 | 35% | 5 | 20% | 18 | 20% | 6 | 24% | 21 | 24% |
| Pallor | 5 | 20% | 8 | 9% | 3 | 12% | 6 | 7% | 4 | 16% | 13 | 15% |
| Dizziness | 5 | 20% | 16 | 18% | 4 | 16% | 11 | 12% | 6 | 24% | 13 | 15% |
| Behavioral change | 4 | 16% | 7 | 8% | 3 | 12% | 4 | 4% | 4 | 16% | 8 | 9% |
| Abnormal head position | 4 | 16% | 6 | 7% | 2 | 8% | 5 | 6% | 4 | 16% | 7 | 8% |
| Drowsiness or fatigue | 4 | 16% | 19 | 21% | 3 | 12% | 14 | 16% | 6 | 24% | 30 | 34% |
| Loss of appetite | 4 | 16% | 12 | 13% | 4 | 16% | 5 | 6% | 4 | 16% | 13 | 15% |
| Weight loss | 4 | 16% | 8 | 9% | 2 | 8% | 6 | 7% | 5 | 20% | 16 | 18% |
| Seizures | 4 | 16% | 6 | 7% | 4 | 16% | 5 | 6% | 4 | 16% | 6 | 7% |
| Other symptoms, not specified | 4 | 16% | 6 | 7% | 4 | 16% | 8 | 9% | 5 | 20% | 1 | 1% |

*Table S2: Presenting symptoms at onset, at first contact to an HCP and at time of diagnosis, shown by COVID-cohort and a historic cohort diagnosed 2015-2019. HCP: Healthcare professional.*

Supplemental table S3: Sub analysis of delay and hesitation in contact to HCPs during COVID-19.

|  | All patients | | Female | | Male | | 0-4 years | | 5-9 years | | 10-17 years | | Low grade | | High grade | | Infratentorial | | Supratentorial | | Spinal | | |
| --- | --- | --- | --- | --- | --- | --- | --- | --- | --- | --- | --- | --- | --- | --- | --- | --- | --- | --- | --- | --- | --- | --- | --- |
| N | 25 | % | 14 |  | 11 |  | 7 |  | 6 |  | 12 |  | 21 |  | 4 |  | 8 |  | 13 |  | 4 |  |  |
| Did you hesitate to contact a doctor due  to the pandemic? |  | |  |  |  |  |  |  |  |  |  |  |  |  |  |  |  |  |  |  |  |  |  |
| No | 22 | 88% | 13 | 93% | 9 | 82% | 6 | 86% | 5 | 83% | 11 | 92% | 19 | 95% | 3 | 75% | 7 | 88% | 12 | 92% | 3 | 75% |  |
| Yes | 2 | 8% | 1 | 7% | 1 | 9% | 0 | 0% | 1 | 17% | 1 | 8% | 1 | 5% | 1 | 25% | 1 | 12% | 1 | 8% | 0 | 0% |  |
| How long did you postpone contact to a  doctor? |  |  |  |  |  |  |  |  |  |  |  |  |  |  |  |  |  |  |  |  |  |  |  |
| 0 days | 12 | 48% | 7 | 50% | 6 | 55% | 3 | 43% | 1 | 17% | 8 | 69% | 11 | 65% | 1 | 25% | 3 | 38% | 9 | 69% | 0 | 0% |  |
| 1-3 days | 2 | 8% | 0 | 0% | 2 | 18% | 0 | 0% | 2 | 33% | 0 | 0% | 1 | 6% | 1 | 25% | 2 | 25% | 0 | 0% | 0 | 0% |  |
| 4-7 days | 1 | 4% | 1 | 7% | 0 | 0% | 0 | 0% | 0 | 0% | 1 | 8% | 1 | 6% | 0 | 0% | 1 | 12% | 0 | 0% | 0 | 0% |  |
| 8-14 days | 1 | 4% | 1 | 7% | 0 | 0% | 0 | 0% | 1 | 17% | 0 | 0% | 1 | 6% | 0 | 0% | 0 | 0% | 0 | 0% | 1 | 25% |  |
| 15-21 days | 1 | 4% | 0 | 0% | 1 | 9% | 0 | 0% | 0 | 0% | 1 | 8% | 0 | 0% | 1 | 25% | 0 | 0% | 1 | 8% | 0 | 0% |  |
| 22 days or more | 1 | 4% | 1 | 7% | 0 | 0% | 0 | 0% | 0 | 0% | 1 | 8% | 1 | 6% | 0 | 0% | 0 | 0% | 1 | 8% | 0 | 0% |  |
| Do not recall | 3 | 12% | 3 | 21% | 0 | 0% | 0 | 0% | 2 | 33% | 1 | 8% | 2 | 12% | 1 | 25% | 2 | 25% | 1 | 8% | 0 | 0% |  |
| How long did you wait for assessment,  from first contact? |  |  |  |  |  |  |  |  |  |  |  |  |  |  |  |  |  |  |  |  |  |  |  |
| 0 days | 8 | 31% | 5 | 36% | 3 | 27% | 0 | 0% | 2 | 33% | 6 | 46% | 7 | 32% | 1 | 25% | 4 | 50% | 4 | 31% | 0 | 0% |  |
| 1-3 days | 5 | 19% | 2 | 14% | 3 | 27% | 2 | 29% | 2 | 33% | 1 | 8% | 4 | 18% | 1 | 25% | 1 | 12% | 1 | 8% | 3 | 75% |  |
| 4-7 days | 3 | 12% | 1 | 7% | 3 | 27% | 2 | 29% | 0 | 0% | 1 | 8% | 2 | 9% | 1 | 25% | 0 | 0% | 3 | 23% | 0 | 0% |  |
| 8-14 days | 2 | 8% | 2 | 14% | 0 | 0% | 1 | 14% | 0 | 0% | 1 | 8% | 2 | 9% | 0 | 0% | 1 | 12% | 1 | 8% | 0 | 0% |  |
| Do not recall | 4 | 16% | 3 | 21% | 2 | 18% | 0 | 0% | 2 | 33% | 2 | 23% | 4 | 18% | 1 | 25% | 2 | 25% | 2 | 15% | 0 | 0% |  |
| How was the initial consultation  conducted? |  |  |  |  |  |  |  |  |  |  |  |  |  |  |  |  |  |  |  |  |  |  |  |
| Consultation by phone | 4 | 16% | 3 | 21% | 1 | 9% | 2 | 29% | 0 | 0% | 2 | 15% | 3 | 14% | 1 | 25% | 2 | 25% | 2 | 15% | 0 | 0% |  |
| Clinical consultation | 14 | 56% | 7 | 50% | 8 | 73% | 3 | 43% | 4 | 67% | 7 | 62% | 13 | 59% | 2 | 50% | 4 | 50% | 7 | 54% | 3 | 75% |  |
| Other consultation | 5 | 20% | 4 | 29% | 1 | 9% | 0 | 0% | 2 | 33% | 3 | 23% | 4 | 18% | 1 | 25% | 2 | 25% | 3 | 23% | 0 | 0% |  |
| Did you experience delays or cancellations  regarding in the early trajectory? |  |  |  |  |  |  |  |  |  |  |  |  |  |  |  |  |  |  |  |  |  |  |  |
| No | 18 | 72% | 11 | 79% | 8 | 73% | 5 | 71% | 5 | 83% | 8 | 69% | 16 | 73% | 3 | 75% | 7$ | 88% | 9 | 69% | 2 | 50% |  |
| Yes | 6 | 24% | 3 | 21% | 3 | 27% | 1 | 14% | 1 | 17% | 4 | 31% | 5 | 23% | 1 | 25% | 1 | 12% | 4 | 31% | 1 | 25% |  |

*Table S3: Sub analysis of delay and hesitation in healthcare professionals, reported by patients diagnosed during the first year of the COVID-19 pandemic.*
